# Flavivirus-Mosquito Interactions

**DOI:** 10.3390/v6114703

**Published:** 2014-11-24

**Authors:** Yan-Jang S. Huang, Stephen Higgs, Kate McElroy Horne, Dana L. Vanlandingham

**Affiliations:** 1 Department of Diagnostic Medicine and Pathobiology, College of Veterinary Medicine, Kansas State University, Manhattan, KS 66506, USA; E-Mails: yshuang1985@ksu.edu (Y.-J.S.H.); shiggs@bri.ksu.edu (S.H.); 2 Biosecurity Research Institute, Kansas State University, Manhattan, KS 66506, USA; E-Mail: kmhorne@ksu.edu

**Keywords:** flavivirus, *Aedes* mosquito, *Culex* mosquito, hemorrhagic fever

## Abstract

The *Flavivirus* genus is in the family *Flaviviridae* and is comprised of more than 70 viruses. These viruses have a broad geographic range, circulating on every continent except Antarctica. Mosquito-borne flaviviruses, such as yellow fever virus, dengue virus serotypes 1–4, Japanese encephalitis virus, and West Nile virus are responsible for significant human morbidity and mortality in affected regions. This review focuses on what is known about flavivirus-mosquito interactions and presents key data collected from the field and laboratory-based molecular and ultrastructural evaluations.

## 1. Introduction to the Flaviviruses

The family *Flaviviridae*, named from the Latin “flavus” for the hallmark jaundice caused by infection with yellow fever virus (YFV), is comprised of the genera *Flavivirus*, *Pestivirus*, and *Hepacivirus*. Mosquito-borne viruses make up a large portion of this family and will be referred to as “flaviviruses” throughout this review. Flaviviruses are encoded by a single-stranded, positive sense RNA genome approximately 11 kb in length. The genome is a single open reading frame encoding 10 viral proteins that are cleaved co- and post-translationally from the polyprotein, the capsid (C), membrane (M), and envelope (E) structural proteins and the nonstructural (NS) proteins 1, 2A, 2B, 3, 4A, 4B, and 5. The polyprotein is flanked by 5' and 3' non-coding regions [1].

As described below, arboviruses within this genus are transmitted by a variety of mosquito species as well as ixodid and argasid ticks. This review will focus on mosquito-virus interactions of four medically important flaviviruses: YFV, dengue virus (DENV) serotypes 1–4, Japanese encephalitis virus (JEV), and West Nile virus (WNV). The geographic distribution of viruses in this family is very broad, and consistent with other arboviruses, the distribution of each virus mirrors that of its vector. It has been estimated that over half of the global population is at risk for infection with one of four dengue virus serotypes (DENV-1, -2, -3, and -4) [2], and YFV, DENV, JEV, and WNV collectively cause millions of infections and tens of thousands of deaths each year [3]. Syndromes following human infection with flaviviruses range from clinically inapparent asymptomatic infections to severe, and sometimes, fatal disease, including hemorrhagic manifestations of severe YFV and DENV infection and encephalitis caused by infection with JEV or WNV. Whereas humans are dead-end hosts for many arboviruses, including JEV and WNV [4], they play a large role in the transmission cycles of YFV and DENV [5,6,7].

## 2. Classification and Evolution

The flaviviruses are subgrouped into nine serogroups, five of which contain important human pathogens. Although mutation rates of up to 10^−4^ substitutions per nucleotide can be attributed to the lack of proofreading capacity of the RNA polymerase, flavivirus evolution is constrained by the need to replicate in invertebrate vectors and vertebrate hosts, as has been demonstrated for the dengue viruses [8]. Based on the host range and choice of vector species, there are four distinct groups: mosquito-borne, tick-borne, no known vector viruses, and insect specific (mosquito only) viruses, with the mosquito-borne group further subdivided into Old World viruses primarily associated with *Aedes* spp. mosquitoes that cause hemorrhagic disease syndromes such as YFV and DENV, Old and New World viruses primarily associated with *Culex* spp. mosquitoes that cause encephalitic disease syndromes, and insect-specific viruses [9,10]. The *Flavivirus* genus has been hypothesized to have emerged from mammalian viruses with no arthropod vector and the mosquito- and tick-borne flaviviruses emerged from Africa [11,12]. This hypothesis is further supported by the phylogeny of flaviviruses showing no known vector viruses are considered ancestral to vector-borne viruses [13,14]. However, the actual emergence of flaviviruses in evolution remains elusive. Proposed theories explaining host preference and vector choice have not been convincing and can further be complicated by the fact that flaviviruses often infect multiple vertebrate hosts and are transmitted by multiple vector species. Earlier ecological studies suggest the narrow host range of flavivirus under the JEV serocomplex is characteristic of being more evolutionarily primitive and the use of a single host by DENV and YFV is a more recent development [15]. Phylogenetic analyses also indicate the more recent emergence of JEV-serocomplex flaviviruses as compared to other viruses under the DENV and YFV serocomplexes [14]. In contrast to mosquito-borne flaviviruses that alternate between vertebrate hosts and arthropod vectors, the group of insect-specific flaviviruses has been demonstrated to evolve and appear in the family multiple times, leading to two major clades associated with *Culex* spp. and *Aedes* spp. mosquitoes [16]. Geographically, the insect-specific flaviviruses co-circulate with pathogenic mosquito-borne flaviviruses. It remains to be seen if the co-infection of insect-specific flaviviruses will change the vector competence of mosquitoes for pathogenic flaviviruses [17].

The prototypic flavivirus, YFV, has evolved into five genotypes, three of which are circulating within the urban cycle in different regions of Africa, and two genotypes are found in Latin America [5,18]. The introduction of YFV into Latin America from Africa likely occurred during the slave trade approximately 300–400 years ago [19]. The sylvatic strains of YFV were distinct from other currently circulating isolates in their growth behaviors in mammalian and insect tissue culture, which is likely reflective of a lack of host adaptation and a difference in ecological conditions [20].

The origin of DENV still remains elusive even though the viruses and ecological systems that support the sylvatic strain of DENV have been discovered both in Asia and West Africa [21]. Historically, the determination of genotypes within each serotype was based on the cut off of 6% of genetic divergence [22]. Currently, there are five genotypes defined under DENV-1 mainly representing the locations of the original isolations [23,24,25]. DENV-2 was comprised of six genotypes, with the Cosmopolitan genotype showing the widest geographic distribution in the majority of the tropical region [26]. DENV-3 and DENV-4 have five and four known genotypes, respectively [27,28,29,30,31,32].

The emergence of JEV was proposed to have occurred in Southeast Asia approximately 350 years ago [33]. There have been four circulating genotypes of JEV recognized with consistent results from different molecular virological tools such as RNase T1 mapping and genomic sequences. Recently, the re-emergence of genotype V was reported in China and Korea following a nearly six decade long absence [34]. With the clusters of genotypes separated by distinct geographic locations, the plausible explanation for the presence of different genotypes in different areas is likely due to the evolution in different mosquitoes and amplification hosts. However, the differences among genotypes I–III are limited to less than 12.0% and 3.5% at the nucleotide and amino acid levels, receptively [33]. Historically, genotype I has been found primarily in Australia, Japan, Korea, Northern Thailand, and Cambodia. Genotype II has been isolated from Southeast Asian countries, especially Malaysia and Indonesia, and was reported to be associated with the first incursion of JEV into Australia in 1995 followed by the isolation of genotype I in Northern Australia in 1998 [35,36]. Genotype III constitutes the largest number of isolates among all genotypes and is found throughout Asia. Genotype IV is ancestral to all circulating genotypes and shows the largest antigenic and phylogenetic differences compared to other genotypes. Although phylogenetic evidence has demonstrated that genotype I has displaced genotype III and become the predominant genotype throughout Asia since the 1990s, there has not been definitive laboratory evidence showing the relative selective advantage of specific genotypes in amplification hosts or arthropod vectors [37].

WNV is another important virus in the JEV serocomplex and circulates in different geographic regions. There are a total of five lineages of WNV which have been discovered, with lineage 1 and 2 having the highest public health significance [38]. Among all five lineages, lineage 1 has the widest geographic distribution and can be found worldwide. In 1999, the introduction of WNV lineage 1 in the United States led to the establishment of New York 99 (NY99) genotype which was found related to an isolate in Israel [39]. As a consequence of continuous evolution, there have been at least three additional genotypes identified in North America: the southeastern Coastal Texas genotype, the North America/WN 2002 genotype, and the Southwest/WN 2003 genotype [40,41,42]. The southeastern Coastal Texas genotype is thought to now be extinct.

Multiple factors can drive the evolution of flaviviruses and create complexity in virus-vector and virus-host interactions [43]. Specific genotypes of the same virus can possess advantages in infectivity, replication, and dissemination in arthropod vectors, which often result in the displacement of other genotypes in nature [37,44,45,46].

## 3. Flavivirus Epidemiology

Mosquito-borne flaviviruses are transmitted in nature in one or more distinct or overlapping cycles that include a mosquito vector, generally *Aedes* spp. mosquitoes for YFV and DENV and *Culex* spp. mosquitoes for JEV and WNV, and a mammalian or avian host. Transmission between mosquitoes and vertebrate hosts is termed horizontal transmissions and causes disease in vertebrates. In contrast to horizontal transmission, mosquito-borne flaviviruses can be maintained in the environment through vertical,* i.e.*, transgenerational, transmissions which allow the spread of flaviviruses solely in mosquitoes [47]. The most direct evidence supporting the vertical transmission of mosquito-borne flaviviruses is derived from the isolation of virus from infected larvae presumably through transovarial transmission [48]. This observation is consistent with the detection of viral antigens in ovarian tissues of infected mosquitoes [49,50,51,52,53].

Both YFV and DENV are transmitted in an urban cycle between humans and *Ae. aegypti*. YFV occurs in enzootic cycles in Africa and the Americas and DENV occurs in enzootic cycles in Africa and Asia. The enzootic cycles of DENV and YFV are mainly maintained between arboreal *Aedes* spp. [54]. In the past decade, epidemics associated with urban cycles of YFV transmission have largely been eliminated in the Americas. However, YFV remains a re-emerging threat because deaths caused by epizootic outbreaks are still reported [55,56,57,58,59,60,61,62]. JEV is widespread across Asia and the Pacific region where it is maintained in an enzootic cycle in Asia between *Culex* spp. mosquitoes and pigs or aquatic birds as amplifying hosts; unlike YFV and DENV, humans are dead-end hosts as they generally do not mount sufficient viremia to infect mosquitoes [63,64,65]. WNV, the most widely distributed of the flaviviruses, is also maintained in an enzootic cycle in affected regions between *Culex* spp. mosquitoes and birds, with humans and horses serving as dead-end hosts [65,66]. The existence of mammalian and avian reservoir hosts for these viruses makes elimination difficult or impossible, so prevention and control must focus on vaccination as well as vector control programs.

Although large YFV epidemics occurred in the 17th, 18th, and 19th centuries and disease reports consistent with YFV date back to the late 15th century [5], the mechanism of YFV transmission was not identified until 1900. Carlos Finlay first proposed a link between YFV and mosquitoes but failed to account for the extrinsic incubation period, the time between uptake of an infectious meal by a mosquito and subsequent transmission by bite, in his evaluations [67]. The Reed Commission composed of Reed, Agramonte, Carroll, and Lazear established the agent that caused YF disease was a filterable agent (not a bacterium or parasite) that was transmitted by mosquitoes [68,69,70]. This discovery substantially impacted public health, particularly in the Americas where the implementation of control programs for *Ae. aegypti* resulted in the elimination of urban YFV and DENV, which shares the same vector [7]. Around the same time, YFV transmission in Francophone Africa was reduced by mass vaccination of human populations with vaccines developed by the French (French Neurotropic Vaccine, FNV) and the Rockefeller YF Commission (17D) through multiple passage of wild-type parental strains through various tissues to derive attenuated viruses [71,72]. Whereas FNV was discontinued in 1971 due to cases of neurotropic disease after vaccination, the YFV 17D vaccine is considered to be one of the safest and most efficacious vaccines available and is still in use today [5]. The only other arbovirus with a vaccine licensed for human use is JEV, which is an inactivated vaccine. Considerable effort has been directed toward the development and testing of live attenuated, recombinant, or inactivated vaccines for DENV and WNV, but none have been licensed for human use. There are no treatments for any of the flavivirus diseases and therapy is mainly supportive. Consistent with other arboviruses, the primary means of control and prevention involves mosquito control, but large mosquito control programs are difficult to maintain, especially in resource-poor countries which are overwhelmingly affected by these diseases. After great success eradicating *Ae. aegypti* from most parts of the Americas, as a result of programs initiated by the Pan American Health Organization, the failure to maintain control efforts resulted in a resurgence of the mosquito and a return of the viruses it vectors [7].

## 4. Selected Medically Important Flaviviruses in Mosquitoes

### 4.1. Yellow Fever Virus

**Figure 1:**
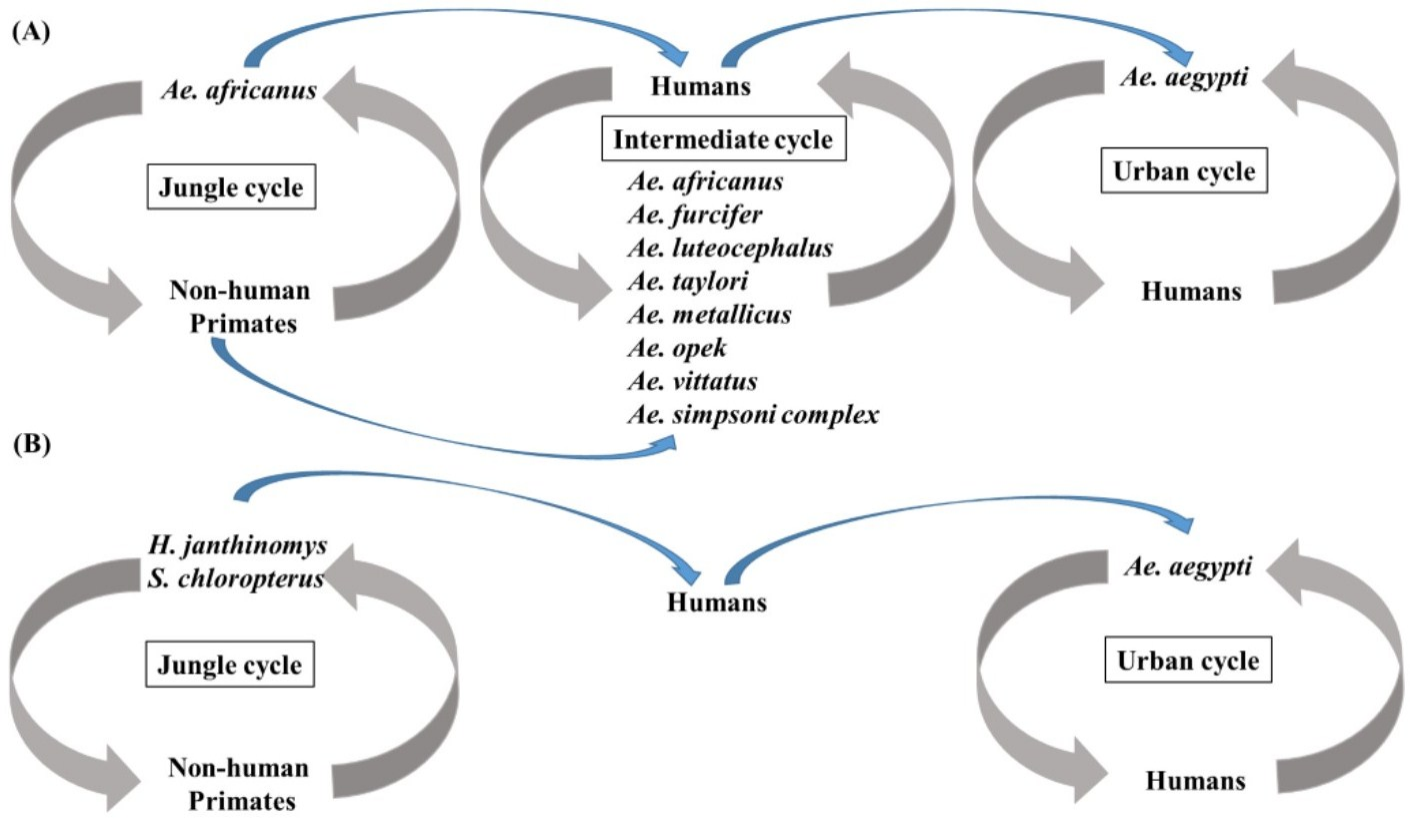
Transmission cycles of yellow fever virus (YFV) in Africa and South America. (**A**) Three transmission cycles support the transmission of YFV in Africa. In the jungle cycle, *Ae. africanus* is responsible for the transmission among non-human primates. In the intermediate cycle, human activities result in the biting of intermediate cycle vectors. In the urban transmission cycle, *Ae. aegypti* transmits YFV by feeding on viremic humans; (**B**) Two transmission cycles of YFV exist in South America. In the jungle cycle, *H. janthinomys* and *S. chloropterus* feed on infected non-human primates. In the urban cycle, *Ae. aegypti* are responsible for virus transmission.

Disease caused by YFV may be subclinical, mild and non-specific, or severe with jaundice, hemorrhage, and death. The first phase of disease, which starts 3–6 days after the bite of an infective mosquito, is non-specific and may include fever, malaise, dizziness, nausea and vomiting, headache, lumbosacral pain, and myalgia. The remission phase either proceeds recovery or may be followed by a period of intoxication characterized by headache, vomiting, jaundice, enlargement of the liver and hemorrhage [6]. Greater than 90% of cases occur in sub-Saharan Africa [73], where the virus exists in a jungle cycle featuring *Ae. africanus*, an urban cycle featuring *Ae. aegypti*, and an intermediate sylvatic cycle that links the two in which tree-hole breeding mosquitoes such as *Ae. africanus*, *Ae. bromeliae*, *Ae. luteocephalus*, *Ae. furcifer*, *Ae. metallicus*, *Ae. opek*, *Ae. taylori*, *Ae. vittatus*, *Ae. simpsoni*, and *Ae. kenysesis* transmit virus to humans and non-human primates [6,73,74]. In South America, the jungle cycle is propagated by *Haemagogus janthinomys* and *Sabethes chloropterus* mosquitoes and humans and non-human primates [6], and urban transmission of virus to humans by *Ae. aegypti* only occurs sporadically. The transmission cycles in Africa and South America are summarized in Figure 1. The virus is absent in Asia and Australia, although mosquitoes in both regions are susceptible to the virus [75,76].

The vast majority of what is known about YFV interactions with mosquitoes has come from studies of the virus in its primary vector, *Ae. aegypti*. The extrinsic incubation period is 9–12 days; however higher temperatures result in more rapid dissemination of virus which shortens the incubation period [77]. Experiments conducted by Davis and Shannon (1928) determined infectious YFV was present in all three mosquito body sections before transmission occurred, including the midguts, hindguts, legs, salivary glands, and ovaries. Later experiments confirmed YFV was able to be transmitted transovarially at a very low rate with only 0.2% of eggs infected [78,79]. Even at this low rate, transovarial transmission may allow virus survival in unhatched eggs during dry or cool periods [6,73]. Other studies found mosquito mortality was not higher in YFV-infected as compared to uninfected *Ae. aegypti* which indicates the virus does not have a deleterious effect on the mosquito. Other experiments found mosquitoes could be infected in the laboratory as long as 110 days after emergence and transmit YFV up to 128 days after infection [80]. In *Ae. albopictus* examined by immunofluorescence assay, YFV was first detected in the posterior midgut, followed by the brain, fat body and salivary glands, but was never detected in the ovaries [81]. YFV tissue tropisms examined by immunohistochemical staining of sectioned *Ae. aegypti* found wild-type YFV infection in the anterior and posterior midgut, cardia, fat body and nervous tissues in all three segments, and salivary glands of *Ae. aegypti* within 14 days of infection, whereas infection of *Ae. aegypti* with the attenuated 17D vaccine strain was limited to the midgut [50].

Several studies reported the ability of *Ae. aegypti* to transmit YFV varied by geographical location [75,82,83], likely due to differences in vector genetics due to population isolation. Wallis* et al.* were able to select for YFV susceptibility by inbreeding isofemale lines of *Ae. aegypti*, and Miller and Mitchell selected for highly susceptible and highly refractory *Ae. aegypti* populations by selective breeding, confirming the role of vector genetics in virus susceptibility [83,84]. This could potentially have implications for control, if the vector genetic factors involved in virus infection and transmission are identified and used to engineer virus resistant mosquitoes. Another potential mechanism for control is the use of the endosymbiotic bacterium *Wolbachia*, as a 10^4^ times reduction in YFV copy number was observed in *Ae. aegypti* with the wMelPop strain compared to control mosquitoes. No difference was seen, however in YFV infection, dissemination, and replication in *Ae. aegypti* with the wMel strain of *Wolbachia* [85], so clearly this avenue requires additional research.

The role of viral genetics in transmission has also been studied extensively for YFV. The FNV virus derived from the French viscerotropic virus is also not transmitted by *Ae. aegypti*, and numerous sequence differences have been identified between the viruses although the contribution of specific amino acids is unknown [71,86]. An additional mutant produced by passage of YFV Asibi in HeLa cells was not transmitted by *Ae. aegypti*, and sequence comparisons revealed 10 amino acid differences from Asibi, including a mutation at position 95 in NS4B that was also present in 17D and FNV [87]. Similarly, infection of *Ae. aegypti* by the YFV 17D vaccine strain has been confirmed in multiple studies to be limited to the midgut [88,89,90,91]; the barrier to transmission has been found to be at the level of midgut escape, as 17D infects the salivary glands at a rate of 100% when the virus is inoculated into the thorax which bypasses the midgut [88,89]. This is most likely the result of a combination of 33 amino acid substitutions accumulated throughout the 17D genome during multiple passage of the wild-type Asibi parental strain. Using Asibi/17D chimeric infectious clones, McElroy* et al.* determined YFV dissemination within *Ae. aegypti* was under multigenic control and the cell receptor binding domain III of the envelope protein, NS2A, and NS4B likely play important roles in productive YFV-*Ae. aegypti* interactions based on chimera phenotypes in infected mosquitoes [89,92,93]. More recent work by Huang *et al.*, found that a substitution at position E-380 modulated YFV infection but not dissemination in *Ae. aegypti* [94]. Additional characterization of the specific amino acids that play a role in YFV infection and dissemination in *Ae. aegypti* is needed.

### 4.2. Dengue Virus Types 1–4

Dengue viruses are often considered the most important arboviruses worldwide causing an estimated 50–100 million infections per year and posing a threat to 2.5 billion people in tropical and subtropical regions. Although the majority of infections lead to self-limited febrile illness, the severe form of the disease can lead to life-threatening dengue hemorrhagic fever which has a mortality rate of 2.5% [95]. Urban transmission of all four serotypes of DENV is mainly mediated by *Ae. aegypti* and *Ae. albopictus* mosquitoes. Over the years, DENV has changed substantially. In the 1970s, only nine countries were considered endemic for DENV, currently there are more than 100 countries in Southeast Asia, Latin America, and the Western Pacific region affected by DENV [96]. Recently, local transmission of DENV has become a re-emerging threat to the United States with reports of local transmission in Florida and Houston, Texas [97,98]. Interestingly, local transmission of DENV in Houston has been shown to occur as early as 2003 [98]. Experimental infection and transmission of DENV in *Ae. aegypti* and *Ae. albopictus* have been extensively performed since the 1970s. Several studies have demonstrated competent vectors of DENV are present in various geographic locations [99,100,101]. Immunostaining of orally infected mosquitoes indicate replication and tropisms of DENV are similar to other flaviviruses [50,51,53,102,103]. The use of reverse genetics systems based on cDNA infectious clones to identify the genetic loci that determine the phenotypes of DENV in mosquitoes was an important advancement [104]. From these studies, there was evidence to suggest there are multiple molecular determinants located throughout the viral genome. The molecular hinge region between domain I and domain II in the envelope protein is likely to control the viral infectivity in *Ae. aegypti* [105]. The FG loop of domain III, which was initially determined to be a receptor-binding region, also contains the critical residues which determine viral infectivity [106,107]. In addition, the rational design of DENV vaccine candidates also identified several critical regions for viral infectivity. The deletion of the 3' untranslated region of DENV-4 resulted in the loss of viral infectivity and restricted replication in the midgut [108]. Recently, mutations in the DENV-2 2'-*O*-methyltransferase were also shown to significantly reduce the infection and dissemination rates in orally challenged *Ae. aegypti* [109].

During the last two decades, significant progress has been made in the understanding of how mosquitoes respond to DENV infection and in the development of genetically modified mosquitoes that are resistant to DENV. One of the major advancements is the more detailed characterization of DENV infection in the arthropod vectors. The evidence demonstrated that innate immunity is an important physiological component to suppress the viral replication in mosquitoes. The expression of innate immunity-related genes in multiple signaling antiviral pathways such as the Toll, JAK-STAT and RNAi pathways can be up-regulated after the ingestion of viremic blood meals [110,111,112,113]. Interestingly, infection in mosquitoes also causes down-regulation of the immune deficiency (IMD) signaling pathway [114]. The suppression of the IMD pathway results in reduced production of antimicrobial peptides, which have been found to be critical for the control of gram-negative bacterial infections in mosquitoes and other insects [115]. Therefore, the immunomodulatory function of DENV can selectively inhibit the immunity that targets other pathogens invading the arthropod vectors such as bacteria. Such immunomodulation was subsequently found advantageous for viral infection as the presence of bacteria in the midgut can cause the activation of immune responses that suppress viral replication [116]. This finding, as well as transcriptome analyses in *Ae. aegypti* challenged by WNV and YFV, suggest that DENV and other flaviviruses actively suppress the expression of antiviral genes that limit viral replication [117]. Although transcriptome analyses of susceptible mosquito strains infected by DENV provided knowledge of how mosquito vectors respond to DENV infection, a critical gap in knowledge exists in the understanding of the variation in vector competence among different vector populations in nature. Transcriptome analyses on susceptible and refractory strains of *Ae. aegypti* suggests the expression of genes associated with specific pathways for metabolism, can contribute to the susceptibility and the refractoriness of *Ae. aegypti* to DENV within 24 h after engorgement of a viremic blood meal [118]. It is also clear the level of activated immune responses is involved in the susceptibility of *Ae. aegypti* to DENV [119]. Another factor required for the establishment of DENV infection in *Ae. aegypti* is V-ATPase which has been identified in RNAi screening and transcriptome analysis and tested with chemical inhibition assays* in vivo* [120,121,122]. Transcriptome analyses have expanded the understanding of mosquito responses to DENV infection. However, significant gaps still exist since the comparison of different serotypes and genotypes of DENV remains limited. With the evidence that different genotypes of DENV-2 show different phenotypes in mosquitoes, viral/vector characterization using reverse genetics systems and mosquitoes with different susceptibility is needed to determine the mechanisms that control the competence of arthropod vectors for DENV [46].

In spite of advancements in the development of vaccines and antiviral therapies, DENV control relies heavily on the reduction of competent vector populations in nature. Several innovative approaches have produced lines of mosquitoes that are refractory to DENV. Combining the viral genetic sequences and the RNAi pathway in mosquitoes, the susceptibility of transgenic *Ae. aegypti* was significantly reduced [123,124]. However, this approach was challenged by the potential loss of resistance in established colonies of transgenic mosquitoes [125]. In addition to engineering mosquitoes that are resistant to DENV, it is possible to engineer mosquitoes using the sterile insect technique (SIT) as another approach for vector control. SIT reduces the total population of competent vectors in disease endemic regions. Mating between a sterile male and a female will not lead to the production of offspring; therefore, by releasing a large number of sterile male insects that compete with normal males for mating, the total mosquito population is decreased. The remarkable success of SIT was first used in eliminating the New World screwworm and has also been applied to the development of genetically engineered *Ae. aegypti* that carry the lethal gene. The introduction of the lethal gene by releasing the transgenic males was expected to reduce the vector population in nature and provide an additional biological control strategy for *Ae. aegypti* [126]. Another method which was considered promising for vector control to reduce the transmission of DENV uses the obligatory intracellular *Wolbachia* to shorten the life span and reduce vector competence of *Ae. aegypti* [127]. Additionally, mating between *Wolbachia*-infected males with uninfected females causes embryonic lethality, which reduces the probability of producing large numbers of offspring which can further sustain the transmission of DENV and other arboviruses transmitted by *Ae. aegypti*. In summary, research on genetically modified *Ae. aegypti* and infection of *Ae. aegypti* by *Wolbachia* demonstrates the knowledge in virus-vector interactions can ultimately be applied to the development of biological control strategies for disease vectors. The development of biological control strategies for *Ae. aegypti* has resulted in the substantial advancement by offering alternative strategies for disease control and prevention. However, the importance of efficacious vaccines and antiviral therapies targeting DENV still should not be ignored.

The development of cDNA infectious clones and molecular genomic tools has improved our understanding of viral and host factors which play a role in the establishment of DENV infection of mosquitoes. The application of such knowledge has been applied for generating vaccine candidates that are biologically non-transmissible by susceptible vectors. Particularly important successes are the Chimerivax^®^-DEN vaccines, which have been showed to impair infection, replication, and dissemination in field-collected *Aedes* spp. mosquitoes [128]. The trivalent Chimerivax^®^-DEN vaccines show promise for the prevention and control of DENV infections. Ultimately, blocking DENV transmission will require a multi-disciplinary approach which targets immunologically naïve populations and competent vectors.

### 4.3. Japanese Encephalitis Virus

**Figure 2:**
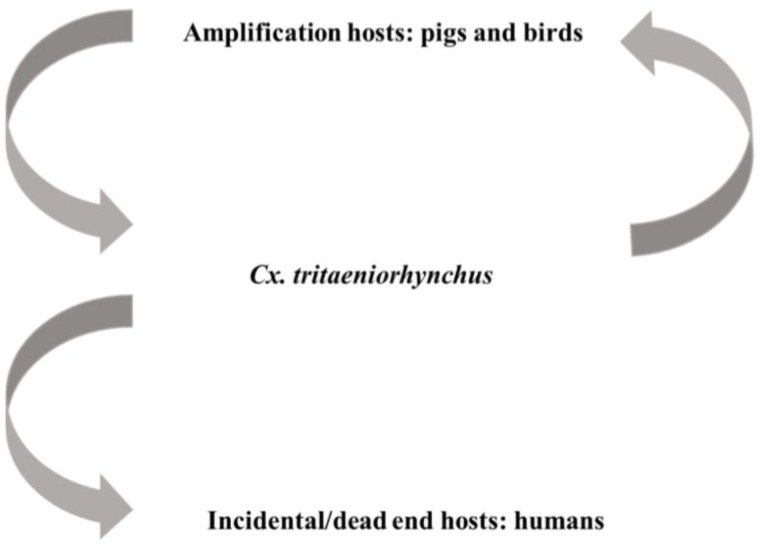
Transmission of Japanese encephalitis virus (JEV) is mainly maintained between viremic amplification hosts and *Culex* mosquitoes, especially *Cx. tritaeniorhynchus*. Infection of incidental hosts, such as humans, are unable to cause high titer viremia and sustain the transmission.

There are approximately three billion humans are at risk of contracting JEV via the bite of *Culex* mosquitoes, especially *Cx. tritaeniorhynchus* and *Cx. vishnui* which are associated with rice farming in Asia [129,130,131]. As shown in Figure 2, transmission of JEV is sustained between avian and swine species that act as amplification hosts and mosquitoes; humans and equine species are incidental hosts in this cycle. Serological surveillance has demonstrated transmission of JEV can be sustained between mosquitoes and swine species without initiating encephalitis outbreaks in humans [132,133]. According to World Health Organization data, there are 30,000–50,000 clinical cases reported annually with a 5%–30% case fatality rate [134]. Approximately 30%–50% of surviving patients experience permanent neuropsychiatric sequelae which creates significant challenges to long-term healthcare and results in severe economic loss. Therefore, several live-attenuated or inactivated vaccines have been developed and are now available for the control and prevention of JEV [63].

The primary vectors of JEV are *Culex* spp. mosquitoes in the Vishnui and Sitiens subgroups. In the Vishnui subgroup, *Cx. tritaeniorhynchus* and other mosquitoes are the competent vectors for transmission in Southeast Asia. JEV emergence has been reported in regions close to the Torres Strait in Australia since 1995 where JEV positive *Cx. annulirostris* and other members of the Sitiens subgroup have been found in nature [135,136,137]. Such epidemiological investigation on JEV-infected mosquitoes in Australia demonstrated the *Culex* spp. mosquitoes under the Sitiens subgroup, which utilize stagnant water as breeding sites, can also be vector species in addition to *Cx. tritaeniorhynchus* and other Vishnui subgroup members that are mainly associated with rice farming [138]. The subsequent evaluation of susceptibility in *Cx. sitiens* collected from Malaysia suggested the same species present in Southeast Asia can also be infected by JEV [139]. In addition to the *Culex* spp. mosquitoes characterized as competent JEV vectors, it is noteworthy that JEV has a relatively wide range of susceptible vector species as several natural isolates of JEV were also reported in *Anopheles* and *Aedes* spp. mosquitoes [140,141,142,143,144]. Another interesting ecological observation was made through the analyses of feeding patterns which reflect the zoonotic nature of JEV. Analyses of feeding patterns of field-collected competent JEV vectors indicate *Cx. tritaeniorhynchus* and* Cx. annulirostris* have tendencies to feed on large mammals. Approximately 10% of the engorged mosquitoes contain blood from pigs. Pigs have been shown to develop a high titer JEV viremia able to cause infection of mosquitoes [145,146,147].

Transmission experiments, using artificial viremic blood meals or through feeding on viremic animals in the laboratory, have identified mechanisms of infection and dissemination of JEV in mosquitoes [53,103,148,149]. One factor that is important for viral transmission is the length of the extrinsic incubation period (EIP) in the mosquito which is determined by the viremic concentration ingested. JEV has been found to be very infectious to mosquitoes with less than 10 plaque forming units (p.f.u.) of virus required to infect competent vector species [150]. Viremic blood meals which were orally administered to *Cx. gelidus* at 10^3.5^–10^4.8^ p.f.u./mL were found to shorten EIP to 6–10 days in contrast to 11–15 days among the mosquitoes exposed to blood meals at 10^1.5^–10^3.4^ p.f.u./mL [149]. During the EIP, the propagation of viruses led to the escape of progeny virions from the midgut and to secondary tissues as visualized by immunofluorescence staining of infected mosquito tissues [53,103]. A consequence of viral replication in the salivary glands is the accumulation of mature virions in the intracytoplasmic region of cells that results in the release of infectious virions into the apical cavity for transmission [148]. In addition to the ingestion of viremic blood meals, the infection of adult mosquitoes can be the consequence of transovarial transmission, which has also been reported as a strategy that allows the maintenance of several other arboviruses in nature [151,152].

Similar to other arboviruses, JEV genetics play a critical role in infection and dissemination in mosquitoes. The attenuated JE 2-8 strain of JEV derived from the serial passage of the virulent SA14 strain* in vitro* resulted in the accumulation of mutations and the loss of infectivity and disseminating capacity in *Cx. tritaeniorhynchus* [153]. In the same study, propagation of the SA14 strain constantly led to the higher titers in either intrathoracically inoculated or orally challenged mosquitoes. However, individual genetic mutations resulting from serial passage* in vitro* that are responsible for the phenotypic changes have not been identified. Additionally, it is unknown if the live-attenuated SA14-14-2 vaccine strain, which was derived from the same parental virulent strain through the serial passages in different cell types, is biologically non-transmissible by mosquitoes. In a study evaluating the ability of the chimeric JEV vaccine to be transmitted by viremic vaccinees found the live-attenuated chimeric JEV vaccine based on YFV backbone did not infect or disseminate in *Cx. annulirostris*, *Cx. gelidus*m, and *Ae. vigilax* [154]. However, it is unclear whether the attenuation is caused by the specific genetic mutations or the chimerization of JEV and YFV genetic materials. As previously reported, the chimerization of genetic materials between two different flaviviruses often resulted in attenuation [89,155]. In contrast to the success in utilizing infectious clones to study the genetic determinants of YFV infection and dissemination in *Ae. aegypti*, the development of JEV infectious clones has been challenging because the propagation of the full-length cDNA genomes in bacterial plasmids often results in rapid mutations [156]. Therefore, the development of cDNA infectious platforms of JEV was either based on multi-plasmid platforms or optimized by the introduction of intron sequences to reduce the toxicity to bacterial hosts [156,157]. Recently, there have been several studies using either low-copy plasmids or bacterial artificial chromosomes in order to maintain the stability of viral genomic sequences in the plasmids propagated in *E. coli* [158,159]. An additional alternative approach has been proposed and found feasible by creating silent mutations by substituting the nucleotide sequences of viral cDNA that resemble the *E. coli* promoter sequences without changing the amino acid sequences [160]. In order to identify the genetic determinants for viral infectivity and disseminating capacity in mosquitoes, an optimized molecular virology platform is needed for the subsequent manipulations of viral genomes. As described above, the major difficulty in developing JEV reverse genetics systems is due to difficulties associated with genetic stability. Without a reverse genetics system that assures the fidelity of cDNA propagation, the evaluation of specific genetic mutations can be technically infeasible. Characterization of viral genetic factors that govern the infection and dissemination of JEV in mosquitoes is critical for the vaccination with live-attenuated vaccines because previous studies have shown infection of arthropods by live-attenuated arbovirus vaccines can potentially contribute to further transmission [161].

Previous studies suggest JEV introduction into new geographic areas is possible where there are competent mosquitoes and susceptible vertebrate hosts. The introduction of JEV into Australia and WNV into the United States exemplify how rapidly JEV-serocomplex flaviviruses can establish in geographic regions where the competent vectors are present and the vertebrate hosts are immunologically naïve. Based on a series of experiments performed with colonized American mosquitoes exposed to JEV, several species have been found susceptible after the engorgement of viremic blood from infected mice [162]. Among the vector species tested for JEV in the study, *Cx. pipiens*, *Cx. quinquefasciatus* and *Cx. tarsalis* where found to be competent for JEV and therefore could potentially transmit this virus in the New World. These mosquitoes are also considered competent vectors for WNV, as discussed below. The large number of farmed pigs combined with potential wild mammal and avian hosts could serve as amplification hosts for JEV after an introduction into the US. In the absence of a comprehensive assessment of vertebrate host and mosquito susceptibility, it is difficult to estimate the likelihood that JEV could potentially become endemic in the US after an introduction.

### 4.4. West Nile Virus

Another medically important flavivirus in the JEV serocomplex is WNV, first isolated from the blood of a febrile woman in the West Nile district of Uganda in 1937 [163]. It is the most widely distributed of the flaviviruses, with strains from one of the two WNV lineages distributed throughout Southeast Asia, southern and Eastern Europe, Australia, and recently, the Americas [164,165,166,167]. Typically arboviruses infect a limited number of vectors with transmission cycles often involving just two or three key species. WNV is unusual since it has been reported to be infectious to more than 60 species of vectors. The establishment of WNV in North America after its introduction in 1999 has demonstrated how readily WNV can adapt and establish transmission cycles in different ecological niches with susceptible vertebrate hosts and competent vector species [168,169,170]. In the Americas, naïve avian populations which lack herd immunity against WNV act as the primary amplification hosts in the transmission cycle [171]. *Culex* spp. mosquitoes are the primary vector in North America and also serve as a bridge vector. Due to the relatively large number of olfactory receptors in *Culex* spp. mosquitoes, they often feed on both viremic avian amplifying hosts and incidental human or equine species [172]. Viremic transmission of arboviruses by mosquitoes requires vertebrate hosts that develop high viremic titers. However, WNV transmission has also been found to occur following the simultaneous feeding of infected and uninfected arthropods on the same animal. Detection of virus is difficult during the initial feeding of an infected mosquito on a vertebrate host; therefore this mode of transmission has been termed “non-viremic transmission”. However, it might more accurately be termed non-replicative transmission because it involves transmission of virus between arthropods without the requirement of host infection and virus replication in the vertebrate [173,174,175]. Transmission takes place primarily due to a transient secretion of a high concentration of virions from infected mosquitoes. The experimental evidence supports such a hypothesis, as the spatial and temporal proximity of feeding between the infected donor mosquitoes and the uninfected recipient mosquitoes are critical factors for the transmission to occur [176]. These findings challenge the established concept that mosquitoes feeding on dead-end hosts cannot contribute to the transmission of WNV.

Several new genotypes have been identified since the establishment of WNV in North America, some of which have been well-characterized. The first genotype to be identified in North America was found in New York in 1999 (NY99). The NY99 genotype was isolated between 1999 and 2003 and has subsequently been considered extinct, possibly due to the displacement by other genotypes [38]. Four additional genotypes have since been identified in North America: NA/WN02, SW/WN03, MW/WN06, and the now extinct southeastern Coastal Texas. Using a method to determine the relative susceptibility of mosquitoes to infection with various genotypes, variation in the oral infectious dose 50% (OID_50_) was found among different WNV genotypes which were orally fed to *Cx. quinquefasciatus* [177]. Between 2001 and 2004, the displacement of the NY99 genotype with the NA/WN02 genotype demonstrated mutations in the E protein can potentially increase the epidemic potential by shortening the incubation period. Initial evidence suggested the NA/WN02 genotype can be more infectious,* i.e.*, lower required infectious dose, based on its higher infection rate in *Cx. pipiens* than the NY99 genotype [44]. However, the NA/WN02 OID_50_ for *Cx. quinquefasciatus* has no demonstrable difference from that of the NY99 genotype, a selective advantage also potentially exists in the dissemination and transmission process [177]. This hypothesis was later validated by *per os* challenge of *Cx. tarsalis*, the predominant enzootic and bridging vector in the western part of United States, with the NA/WN02 genotype, which had a shorter incubation period than infection with the NY99 genotype [45]. Such a difference was attributed to the E-V159A mutation. Genetic characterizations also demonstrated the advantageous role of the E-V159A mutation, which is conserved among all currently circulating genotypes. The southeastern Coastal Texas genotype, which does not contain the E-V159A mutation, was transiently circulated before 2002 [40]. In addition to phenotypic effects of changes in the E gene, attenuation of WNV can be achieved by manipulating the NS2, NS4B and NS5 genes [178,179,180]. These studies used reverse genetics systems to introduce point mutations to WNV genomes in order to functionally characterize the nonstructural proteins and ultimately lead to attenuated strains of WNV. Originally identified in JEV and further proposed as a potential attenuation mechanism for the SA14-14-2 vaccine strain, the programmed ribosome frame shift occurs in the 5' terminus of the NS2 gene and results in the production of an additional nonstructural protein NS1' in several JEV-serocomplex flaviviruses including WNV [181,182,183]. Although the functions of NS1' proteins have not been completely understood, the abolishment of the production of NS1' has been demonstrated to impair the replication and dissemination of WNV in *Cx. annulirostris* [184]. Mutational analyses of the WNV NS4B protein demonstrated the *N*-terminal and the central hydrophobic regions contain the genetic determinants for virulence in mice [178,179], but such mutations in NS4B, the P38G and T116I substitutions, resulted in the enhancement of viral dissemination and transmission in *Cx. tarsalis* [185]. Evaluation of critical residues in the rNTP-binding region of WNV NS5 protein showed deliberate stabilization of the local secondary structures by increasing the molecular interactions through genetic mutations can result in the loss of viral fitness including the lower level of viral replication in *Cx. pipiens* [180]. Characterization of WNV genotypes found selective advantages such as higher infectivity and shorter extrinsic incubation periods in mosquitoes are due to mutations in the E protein. Molecular virological manipulation of WNV demonstrated several genetic loci in the non-structural genes that can lead to the attenuation of WNV* in vivo*; especially the abolishment in the production of NS1' protein. However, the mutations in the NS4B protein, which were reported to reduce the virulence in mammalian hosts and considered attenuation determinants, showed the opposite impact by increasing the viral fitness of WNV in specific infected mosquito species.

For all genotypes of WNV, maintenance of viral populations through winter climatic conditions is an important ecological question because of the lack of active transmission in the winter. It seems reasonable to speculate WNV may share certain overwintering mechanisms with closely related JEV such as transovarial transmission and persistent infection of diapausing mosquitoes. WNV viral RNA can be detected in hibernating *Culex* spp. mosquitoes in nature, so persistent infection has been identified as a potential mechanism for viral maintenance during cold periods [186]. Although prolonged WNV infection during overwintering can lead to cytopathic effects in mosquitoes, the transmission capacity of persistently infected mosquitoes is unaffected [187,188]. There are a large number of competent vector species for WNV, so it is likely virus overwintering occurs in multiple species. For example, it has been suggested species such as *Cx. restuans,* whose adult populations peak in the spring, serve as the early amplification host for WNV prior to the emergence of other vector species [189]. WNV has been detected in ovaries and neighboring tissues [52] and WNV can be maintained through transovarial transmission in *Cx. pipiens*, *Cx. quinquefasciatus*, *Cx. tarsalis* and *Cx. vishnui* [190,191].

Although infection of WNV has been known to occur in various *Culex* spp. mosquitoes, the detailed characterization of how mosquitoes develop physiological and antiviral responses to WNV infection has only recently been studied since the elucidation of the *Cx. quinquefasciatus* genomic sequence [172]. RNAi antiviral responses described in other arboviruses were found to be important for limiting infection of WNV and subsequently inducing diversification of viral genetics [192,193]. Additional immune signaling pathways can potentially be induced including Toll, Imd, and JAK/STAT signaling pathways [194]. The most direct evidence was derived from the observation that the secreted form of Vago protein is critical for the induction of JAK/STAT signaling and restricting WNV replication* in vitro* [195]. Experiments using *Ae. aegypti* as a model found WNV infection leads to the down regulation of genes which limit the viral replication [117]. This observation is consistent with the immunomodulation caused by DENV in mosquitoes indicating WNV infection may also actively suppress the antiviral responses developed by mosquitoes.

The study of WNV in mosquitoes has been further extended to the characterization of interactions among viruses, mosquitoes, and vertebrate hosts. An interesting discovery resulting from studies designed to characterize interactions between WNV and mosquitoes is the potentiation of vertebrate infection caused by the salivary component of mosquitoes [196]. The presence of salivary components enhance the disease progress of WNV in the murine model and mosquito feeding increased mortality rates among infected mice [197]. Based on these and other studies, the potentiation of infection is due to the modulation of host immune responses, especially suppressing the antiviral T_H_1 immune response, and other factors, for example migration of susceptible cell types to the inoculation site [198].

Observations from the relatively short time since the introduction of WNV into the Americas and its continued spread suggests further genetic changes may occur in response to additional selection pressure. Because WNV is principally transmitted by multiple *Culex* spp. mosquitoes, it remains controversial if specific genetic substitutions that favor the establishment of infection in a particular species also have a similar effect on WNV infection in other mosquitoes [199]. Previous studies predominantly focused on evaluating the susceptibility and the capacity of WNV transmission by particular species; future investigations of the temporal and spatial patterns of viral infection of WNV vectors may characterize viral or vector genetic factors that determine ecological selective advantage or relative disadvantage of specific genotypes compared to others under certain conditions.

## 5. Conclusions

There are still significant gaps in the fundamental knowledge related to many arboviruses. Despite physical geographic boundaries, the distribution of specific flaviviruses has continuously changed throughout human history. It is intuitive to assume the presence of competent vectors and susceptible hosts in suitable climatic conditions are sufficient for the introduction of flaviviruses into areas where they are originally absent. For example, YFV was introduced and readily established its transmission in the New World because of the slave trade [19]. More recently, the eastward spread of JEV which successfully crossed the Torres strait and crossed the hypothetical Wallace line, that separates Southeast Asia from Australia, in late 20th century [136]. Similarly, despite the large distance from endemic areas, there have been abrupt and explosive epidemics of WNV in North America since 1999 [129]. In the latter two examples, the establishment of transmission cycles was a consequence of flaviviruses vectored by mosquito species which are different from the original endemic regions. Therefore, it is immediately apparent that the knowledge in flavivirus-mosquito interactions is critical for the prediction of the changing epidemiology and the epidemic potential of mosquito-borne flaviviruses. Clearer understanding of the vectors and vertebrate host interactions may enable predictions of how these viruses can spread from one region to another in the future and ultimately be applied to the development of more efficient disease control strategies.
